# Impact of an Educational Measure on the Quality of Medical Certificate of Cause of Death at a Tertiary Care Teaching Hospital: A Pre-post Interventional Study

**DOI:** 10.7759/cureus.54721

**Published:** 2024-02-22

**Authors:** Aashish Chaudhary, Gaurav Singh, Abhishek Hooda, Jyoti Garg, Divya Saharan, Surendra Kumar, Karnika Agrawal, Nazir A Pandit

**Affiliations:** 1 Paediatrics, Maharaja Agrasen Medical College, Agroha, Hisar, IND; 2 Community Medicine, World College of Medical Sciences Research and Hospital, Jhajjar, IND; 3 Hospital Administration, Maharaja Agrasen Medical College, Agroha, Hisar, IND

**Keywords:** mortality data, cause of mortality, pre- and post-interventional study, educational intervention, death audit, medical certificate of cause of death, death certificate

## Abstract

Background

A death certificate is an important document that serves as a tool for gathering epidemiological data and as an essential legal document. Although it is a mandatory document to be given for all deaths, the quality of its filling is often an ignored aspect and errors are frequently encountered. This documentation process can be mastered with minimal educational efforts. This study aimed to determine the utility of an educational measure in improving the accuracy of death certificate documentation.

Methods and materials

This pre- and post-interventional study was conducted at Maharaja Agrasen Medical College, Agroha, a tertiary care teaching hospital in Hisar, Haryana, India, wherein an audit of death certificates was done before and after an educational intervention on doctors responsible for filling death certificates. Errors in the death certificates were classified into major and minor errors and compared in the pre- and post-intervention groups.

Results

A total of 184 pre-intervention and 136 post-intervention death certificates were audited. In the pre-intervention certificates, at least one major and one minor error were present in 88% and 92.93% of the certificates, respectively, which was reduced to 33% (p < 0.01; relative risk (RR) = 3.62; 95% confidence interval (CI) = 2.69-4.91) and 38% (p < 0.01; RR = 3.33; 95% CI = 2.53-4.37), respectively, post-intervention. Reduction in all types of major and minor errors was statistically significant (p < 0.05).

Conclusions

Errors in death certification are a common but frequently ignored problem that can have a negative impact on epidemiological data and can be drastically reduced with simple educational measures, which need to be carried out regularly.

## Introduction

A government-issued official document, the death certificate, provides information regarding the cause, location, and time of death, along with various personal details about a deceased individual. Issuing of death certificates in India is mandatory as per the Registration of Births and Deaths Act of 1969 [1,2]. The completion of a death certificate is a routine aspect of medical care. A death certificate functions as a tool for gathering epidemiological data and information and a legal document for the deceased’s family. The role of a medical officer is not only to declare a person dead but also to issue a medical certificate of cause of death (MCCD). The same medical officer who proclaimed the deceased dead also handles the second task of producing an MCCD.

A cause of death refers to a disease, abnormality, injury, or poisoning that had a direct or indirect role in the person's demise. The underlying cause of death can be defined as the disease or injury that initiated the sequence of pathological events directly leading to death or the conditions of an accident or act of violence that resulted in the fatal injury. All the morbid conditions or injuries that follow as a result of the underlying cause leading to death are collectively referred to as antecedent causes, while the terminal event is called the immediate cause [1].

An MCCD consists of part 1 and part 2. Part 1 consists of three lines, designated as a, b, and c. Each line should contain only one cause of death. The uppermost line "a" denotes the immediate cause of death, line "b" denotes the antecedent cause of death, while the lowest line "c" represents the underlying cause of death. If there are more than three stages in the sequence, additional lines can be added between the underlying cause of death and antecedent causes of death. Hence, accordingly, the most recent cause of death should be in the first line (line a), while the underlying cause of death should be in the last line. If there are only two causes in the sequence of death, the lowermost line, for example, line b, will present the underlying cause of death. There may be multiple sequences of events in the death process, but the most appropriate sequence of events will be entered in the death certificate. Part 2 contains contributing causes of death in which we are required to write other significant conditions contributing to the death but not causally connected to the cause of death. There can be multiple contributing causes in part 2. The other required information in the death certificate includes the name of the hospital, ward, and the deceased’s sex, age, manner of death (whether natural or unnatural), and pregnancy status [1,3].

Accuracy in certifying the cause of death is desirable to provide reliable information to health planners and families of the deceased and to assess the performance of individual doctors. However, studies have shown that death certificate error rates are quite high, even in teaching hospitals [3]. Improper filling of a death certificate can lead to erroneous epidemiological data and misguided allocation of national resources due to false over-reporting of certain causes of death, such as cardiovascular causes [3]. Errors in filling up the death certificate are common problems in India and the world [4-14]. This is because only a few institutions or residency programs in India provide formal training in the death certification process. Almost all doctors fill out death certificates, but many express doubts regarding the right procedure for doing so. Errors can happen at many stages of the certification process leading to inaccurate death certificate information. Inadequate clinical information can lead to an erroneous antemortem diagnosis, which also reflects in death certification errors. Since there is a need to improve the accuracy of death certificates, this study aimed to determine the impact of educational interventions on the quality of death certificate completion.

The study plan was selected for undergraduate student scholarship by the Indian Council for Medical Research, and the final report was submitted in 2022. The paper was also presented at the State Conference of the Indian Academy of Pediatrics, Haryana, in May 2023.

## Materials and methods

This hospital-based pre-post interventional study was conducted in the Pediatrics, General Medicine, and Emergency Departments of Maharaja Agrasen Medical College, Agroha, a tertiary care teaching hospital in Hisar, Haryana, India, India. A total of 52 junior residents, postgraduates, and medical officers responsible for filling up death certificates were included as study participants. Before initiating the study, approval was secured from the Institutional Ethics Committee (approval no. MAMC/IEC/2022/35), and written informed consent was obtained from all participating doctors.

**Figure 1:**
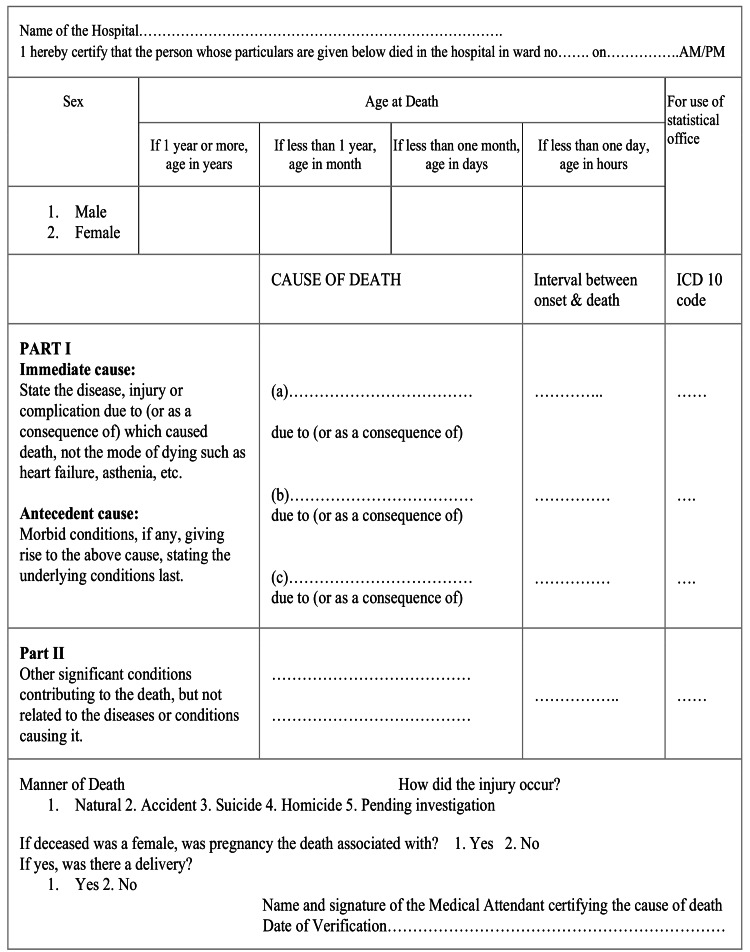
Standard format of medical certificate of cause of death in India for Institutional deaths (form) Adopted from the framework for the audit of medical certification of cause of death at the health facility by the Indian Council of Medical Research (ICMR) - National Centre for Disease Informatics and Research [3].

The sample size was calculated by the online, web-based software OpenEpi (version 3), an open-source calculator developed by the Centers for Disease Control and Prevention (CDC). In the study done by Selinger et al., 58.6% of certificates were incorrect, which was further decreased to 20% after an intervention [4]. Considering this incidence in both groups with a 95% two-sided significance level and 95% power, the minimum sample size obtained was 45 in each group. In our study, however, we took 184 death certificates before intervention and 136 death certificates after intervention (total = 320). The standard format of MCCDs in India, adopted from the WHO International Form of Medical Certificate of Cause of Death, was used in the study (Figure 1).

A total of 184 most recent death certificates were obtained from the hospital records section of the last two months and were audited for accuracy, completeness, and frequency of major and minor errors. Then, an educational intervention program was conducted for all doctors preparing death certificates who were trained in groups of five to 10. The training module consisted of an exemplification of erroneous death certificates filled by doctors on previous occasions, a detailed elaboration of mistakes, followed by a short lecture highlighting the importance of death certification, process of filling, and common errors encountered in detail. This was followed by practice sessions with case-based discussions using virtual case scenarios. The whole session lasted about two hours. After this educational intervention to residents and medical officers, another audit of the next 136 death certificates, which were filled up after the intervention in the next two months, was conducted. Death certificates were again analyzed for major and minor errors, as described in Table 1. Pre-intervention and post-intervention errors in filling up of death certificates were identified and recorded in a Microsoft Excel master chart. Categorical variables were summarized as percentages. The rate of errors was noted before and after giving educational intervention, and the decrease in errors was checked for statistical significance by a chi-square test at a p-value <0.05.

**Table 1:** Classification of errors into major and minor

Types of error	Definition
Major errors	
E1: Mechanism listed without cause	Only the mechanism or the nonspecific condition is listed in line a or documented as the underlying cause of death (lowermost line of part 1).
E2: Improper sequencing	Inappropriate order or sequence of events. (Even if the mechanism of death is listed as the immediate cause of death on line a and improper sequence on lines b and c, it is considered as E2 only and not E1.)
E3: Competing causes	Multiple diseases, each with distinct causes and not linked causally, are used in single or separate lines. (Even if the mechanism of death is listed as the immediate cause of death on line a and multiple causally unrelated diseases on lines b and c or any single line, it is considered as E3 only, not E1.)
Minor errors	
e1: Abbreviations	Abbreviations are used instead of full forms.
e2: Absence of a time interval	The time interval is not listed.
e3: Mechanism of death followed by a legitimate underlying cause of death	The mechanism or mode of dying is written as the immediate cause of death but an etiological specific underlying cause of death mentioned correctly.

Major errors are those that lead to a change in the underlying cause of death, resulting in an incorrect attribution. Minor errors, on the other hand, do not alter the underlying cause of death but may involve certain deficiencies.

To illustrate the classification of errors, we consider the following example: A four-year-old boy with a medical history of cerebral palsy and asthma presented in the emergency department with a four-day history of aspiration pneumonia. The patient subsequently developed respiratory failure, leading to acute respiratory distress syndrome, necessitating intubation, and ultimately succumbed. The death certification for this case, conducted by different medical professionals, is comprehensively analyzed in Table 2 to facilitate a clearer understanding of major and minor errors.

**Table 2:** Analysis of the death certificate of index case filled by four doctors

Cause of death	Interval between the onset and death	Error	
Doctor A		The mechanism or the nonspecific condition is documented as the immediate cause of death with no time interval mentioned; however, the underlying cause of death is correct. (E3 and E2)	
Part I	-		
a. Respiratory failure	-		
b. Cerebral palsy	-		
Part II			
Doctor B			Improper sequence and abbreviation are used. (E2 and E1)	
Part I			
a. Aspiration pneumonia	4 days		
b. ARDS	1 day		
c. Cerebral palsy	Since birth		
Part II			
Doctor C		Competing causes: Multiple diseases, each with distinct causes and not linked causally, are mentioned. (E3)	
Part I			
	a. Asthma		1 year	
b. Cerebral palsy	Since birth		
c. Pneumonia	4 days		
Part II			
Doctor D			Correct	
Part I			
a. Acute respiratory distress syndrome	1 day		
b. Aspiration pneumonia	4 days		
c. Cerebral palsy	Since birth		
Part II			
Asthma	1 year		

## Results

A total of 320 death certificates (184 pre-intervention and 136 post-intervention) were examined. Death certificates that were filled during the pre-intervention period were found to be unsatisfactory, and 88.04% contained at least one major error. “Absence of time interval” was the most common type of minor error that was present in almost every death certificate (91.85%). After the educational intervention was done, the rate of errors was reduced significantly.

As shown in Table 3, after the intervention, major errors and minor errors showed a significant drop in all the categories. The most common major error was writing the mechanism of death as a cause of death on any line in part one or two, which was due to unawareness among the doctors and was improved significantly after the educational intervention.

**Table 3:** Comparison of errors in death certificates pre- and post-intervention RR: relative risk, CI: confidence interval

Type of error	Pre-intervention (n = 184)	Post-intervention (n = 136)	p-value	RR (95% CI)
Major errors	N (%)	N (%)		
Mechanism listed without a cause	88 (47.83)	19 (13.97)	<0.01	3.42 (2.20-5.33)
Improper sequencing	42 (22.82)	08 (05.88)	<0.01	3.88 (1.86-7.99)
Competing causes	32 (17.39)	06 (04.41)	<0.01	3.94 (1.70-9.16)
Minor errors				
Use of abbreviations	71 (38.59)	16 (11.76)	<0.01	3.28 (1.99-5.36)
No time interval listed	169 (91.85)	31 (22.79)	<0.01	4.03 (2.94-5.51)
Mechanism listed with legitimate underlying cause of death	27 (14.67)	10 (07.35)	0.04	1.99 (1.01-3.98)
At least one major error	162 (88.04)	33 (24.26)	<0.01	3.62 (2.69-4.91)
At least one minor error	171 (92.93)	38 (27.94)	<0.01	3.33 (2.53-4.37)

## Discussion

Death certification errors are a common occurrence worldwide, and India is not untouched [4-14]. The main purpose of this study was to estimate common errors in filling up death certificates and to determine the impact of educational interventions on reducing these errors. The results of this study have clearly shown that sensitizing the doctors responsible for certifying the cause of death by a simple educational intervention does decrease the rate of errors in filling out death certificates. A significant decline in both major and minor errors was demonstrated in this study.

The quality of death certificates is a much-neglected aspect in India as suggested by various Indian studies, which reveal a higher rate of errors in comparison to non-Indian studies. In our study also, the rate of errors in death certification during the pre-intervention period was considerably higher than those in various non-Indian studies [4-14]. Our study documented at least one major error in 88.04% and at least one minor error in 92.93% of the death certificates filled before the educational intervention. This was similar to the study of Patil et al. [6] who found at least one major error in 87.76 % of death certificates while 99.25% had minor errors. The most common major error found in their study was sequencing errors (64.74%), while in our study, it was “mechanism listed without cause” (47.83%). The most common minor error observed by them was “no mention of time interval” (98.92%), which was similar to our study (91.85%) [6]. Another Indian study done by Azim et al. [7] showed an even higher rate of at least one major pre-interventional error (98.6%) when compared to our study (88.04%). It can be attributed to the general neglect in the documentation process even in teaching hospitals in India.

Various other studies also show that death certificates come out to be more accurate and legitimate as a result of improved education [4-15]. The design of this study was comparable to that of Myers et al. [5] as this study used a similar method for the classification of errors into major and minor with some modifications. We modified the classification slightly to avoid overlapping of any two major errors in a single filled death certificate. This study, though 25 years old, remains relevant due to the widespread ignorance about filling out death certificates correctly. The major error that was most prevalent in our study was “mechanism listed without cause,” which was present in 47.83% of death certificates before the intervention, whereas only 15.8% of certificates analyzed by Myers et al. [5] contained this type of error. After the intervention, this error reduced to 13.97% in our study and to 4.8% in the study done by Myers et al. [5]. This demonstrates that educational interventions are useful in decreasing the major errors in death certificates. The most common minor error in our study was “no time interval listed,” which reduced from 91.85% before the intervention to 22.79% after the intervention, which is quite a significant change. Meanwhile, in the study of Myers et al. [5], the change in the minor errors was not significant. In this study, there was a significant decrease in both major and minor errors, but the study done by Myers et al. showed only a decrease in major errors [5].

Selinger et al. (2007) found 58.6% errors in the death certificates assessed before a simple educational measure, which was reduced to 20% later on [4]. The educational intervention used by them was a much simpler one when compared to ours [4]. Azim et al. (2014) intervened using educational sessions, interactive workshops, and monthly audits for resident doctors and examined 75 death certificates pre- and post-intervention, respectively, for major and minor errors [7] as defined by Myers et al. [5]. They also found a significant decrease in major and minor errors post-intervention. Major errors, such as mechanism without underlying cause of death, decreased from 60.0% to 14.6%, while in ours, it was from 47.83% to 13.97%; competing causes decreased from 88.0% to 13.3%, which was 17.39% to 4.41% in ours; and improper sequencing reduced from 89.3 to 36.0%, while in ours, it was decreased from 22.82% to 5.88%. There was also a significant decrease in minor errors, such as the use of abbreviations (89.3-29.3%) and no time intervals (100.0-22.6%) similar to our study [7].

In the study by Park et al. (2022), 87 death certificates were examined for major and minor errors using the International Statistical Classification of Diseases and Related Health Problems 10th revision guidelines [12]. A high incidence of errors was found (97.5% and 100% in the disease and external groups, respectively). However, they did not carry out any intervention in their study [12]. Wojcik et al. (2022) undertook a quality improvement initiative using Plan, Do, Study, Act tests of change to improve death reports in their NICU, which significantly decreased the proportion of incorrect death certificates from 71% to 22% [15].

This study demonstrates that the quality of death documentation in a teaching hospital in India is often ignored, resulting in documents of poor value that are amenable to appropriate educational interventions. To improve the quality of death certification, it should be ensured that only doctors who had taken care of the patient in their terminal days fill out the death certificates and have a proper education in the filling of death certificates, to be reinforced at regular intervals with training programs. Giving proper educational intervention has positive effects not only in the case of death certificates but also in overall medical documentation.

The limitations of the study were that only filled death certificates were audited without taking into account the clinical course and history of patients, so the diagnosis was not verified. Other significant conditions could also have been added to minor errors, such as errors in ICD10 coding, name, age, sex, hospital name, pregnancy status, and nature and manner of death. One of the strengths of the study is that the classification of major errors was done in such a way that the overlap of major errors was not possible.

## Conclusions

The majority of death certificates issued contain avoidable errors. Death certificate completion is a neglected aspect even in teaching hospitals. Sensitization by simple educational measures has decreased the number of death certificates that do not fulfill legal requirements and the number of minor mistakes and omissions.

All doctors must become proficient in death certification to increase the precision of epidemiological data. Death certification documentation should be included effectively in the curriculum of both medical students and residents so that they are familiar with the documentation process. All physicians must be sensitized to the necessity of proper documentation, and sustained educational efforts should be undertaken to reduce errors. This will vastly improve medical documentation.
